# Polymorphisms of *TGFB1 *and *VEGF *genes and survival of patients with gastric cancer

**DOI:** 10.1186/1756-9966-28-94

**Published:** 2009-06-30

**Authors:** Xiaoxiang Guan, Hui Zhao, Jiangong Niu, Dongfeng Tan, Jaffer A Ajani, Qingyi Wei

**Affiliations:** 1 Department of Epidemiology, The University of Texas M. D. Anderson Cancer Center, Houston, Texas 77030, USA; 2 Department of Pathology, The University of Texas M. D. Anderson Cancer Center, Houston, Texas 77030, USA; 3 Department of Gastrointestinal Medical Oncology, The University of Texas M. D. Anderson Cancer Center, Houston, Texas 77030, USA

## Abstract

**Background:**

Some *TGFB1 *and *VEGF *polymorphisms are believed to be functional. Given that these genes are involved in tumor growth and progression including angiogenesis, dissemination, and invasiveness, we hypothesized that these polymorphisms would be associated with survival in patients with gastric cancer.

**Methods:**

We genotyped *TGFB1 *-509 C>T, +1869 T>C, and +915 G>C and *VEGF *-1498T>C, -634G>C, and +936C>T in 167 patients with gastric cancer. Using the Kaplan and Meier method, log-rank tests, and Cox proportional hazard models, we evaluated associations among *TGFB1 *and *VEGF *variants with overall, 1-year, and 2-year survival rates.

**Results:**

Although there were no significant differences in overall survival rates among all polymorphisms tested, patients with *TGFB1*+915CG and CC genotypes had a poorer 2-year survival (adjusted hazard ratio (HR), 3.06; 95% confidence interval (CI), 1.09–8.62; *P *= 0.034) than patients with the GG genotype had. In addition, patients heterozygous for *VEGF *-634CG also had a poorer 1-year survival (adjusted HR, 2.08; 95% CI, 1.03–4.22; *P *= 0.042) than patients with the -634GG genotype.

**Conclusion:**

Our study suggested that *TGFB1*+915CG/CC and *VEGF *-634CG genotypes may be associated with short-term survival in gastric cancer patients. However, larger studies are needed to verify these findings.

## Introduction

In gastric caner, patients with the same clinicopathologic characteristics and the same treatment regimens may have different clinical outcomes. Although stage is the best available clinical measure of tumor aggression and prognosis, there are clearly important differences even within the same tumor stage [1,2]. Therefore, it would be helpful to improve the prognostic accuracy by identifying readily accessible molecular markers that predict some of the variation in clinical outcomes. In recent decades, many studies have shown that genetic alterations play roles in the development and progression of gastric cancer [3]. Among these molecular markers, single nucleotide polymorphisms (SNPs) are the most commonly investigated genetic variation that may contribute to patients' clinical outcomes [4].

Epidemiologic and clinical investigations have suggested that both TGF-β1 and VEGF may play an important role in the oncogenesis of the stomach [5,6]. For example, *TGFB1 *and *VEGF *variants are associated with altered protein products, which may contribute to variation in individual susceptibility to cancer and clinical outcomes [4]. Both *TGFB1 *and *VEGF *genes are highly polymorphic, reportedly having 168 and 140 variants, respectively, but only few of these variants are within the promoter or coding regions that may be potentially functional . Of these variants, several SNPs have been described as important in modulation of gene functions [7-9] and reportedly involved in the etiology of various cancers [10-13].

The TGF-β1 pathway is critically involved in tumor development and progression. In tumor cell cultures, TGF-β1 has anti-proliferative effects and can block tumor progression in its early stages, whereas it can also accelerates invasion and metastasis in the later stages of tumor progression [14,15]. One experimental study reported that TGF-β1-mediated activation of the ALK5-Smad 3 pathway is essential for the Shh protein to promote motility and invasiveness in gastric cancer cells [16]. Mouse experiments also showed that altered TGF-β1 was associated with the latent TGF-β1 binding proteins that can cause inflammation and tumors [17] and that the disrupted TGF-β1 pathway can lead to tumor growth by increasing the tumor angiogenesis induced by decreased expression of thrombospondin-1 [18]. In humans, TGF-β1 had a greater sensitivity than carcino-embryonic antigens in tumor cells from gastric cancer patients [16]. Furthermore, both experimental and clinico-pathological studies have suggested a role for the VEGF family of proteins in metastasis through the lymphatic system and in clinical outcomes in several human solid tumors, including gastric cancer [19].

In this study, we chose to genotype selected common (i.e., minor allele frequency > 0.05) *TGFB1 *and *VEGF *SNPs that either lead to non-synonymous amino acid changes [20] or have been associated with lower expression levels of these genes [8,21], which imply these SNPs may be functional. We hypothesized that potentially functional polymorphisms in *TGFB1 and VEGF would be associated with clinical outcomes in patients with gastric cancer. Specifically, we *evaluated the association between clinical outcomes in gastric cancer, including overall survival, and each of the following SNPs: three *TGFB1 *SNPs, including one promoter SNP (-509 C>T) and two exon 1 SNPs (+869 T>C and +915 G>C) and three *VEGF *SNPs, including one promoter SNP (-1498T>C), one 5'-untranslated region SNP (-634G>C) and one 3'-untranslated region SNP (+936 C>T).

## Methods

### Study population

This prospective analysis consisted of 167 patients with newly diagnosed and histologically confirmed gastric cancer, who were treated at The University of Texas M.D. Anderson Cancer Center, Houston, Texas between April 2003 and July 2008. The study protocol was approved by our Institutional Review Board (IRB) and all patients gave informed consent using the IRB-approved informed consent form. Exclusion criteria included those not newly diagnosed and those having been treated elsewhere before coming to M. D. Anderson. These patients were included in this analysis because their stored blood samples were available for DNA extraction.

### Genotyping

Genomic DNA was extracted from the buffy coat fraction of the blood sample of each patient by using a Blood Mini Kit (Qiagen, Valencia, CA) according to the manufacturer's instructions. DNA purity and concentrations were determined by spectrophotometric measurement of absorbance at 260 and 280 nm by UV spectrophotometer. The three selected *TGFB1 *SNPs [one (-509 C>T/rs1800469) in the promoter and two (+869 T>C/rs1800470 and +915 G>C/rs1800471) in exon 1] and three promoter *VEGF *SNPs [one (-1498T>C/rs833061) in the promoter, one (-634G>C/rs2010963) in the 5'-untranslated region, and one (+936C>T/rs3025039) in the 3'-untranslated region] were genotyped using polymerase chain reaction(PCR) – restriction fragment length polymorphism (RFLP) method. Genotypes of the *TGFB1 *SNPs were determined as previously described[22], and assays on the *VEGF *SNPs were also previously reported [23]. For the PCR-RFLP-based genotyping assay, two research assistants independently read the gel pictures, and the repeated assays were performed, if they did not agree on the tested genotype. In addition, repeated assays were performed on a randomly selected 10% of the samples were randomly selected to perform the repeated assays with the results being 100% concordant.

### Outcome data collection

All 167 gastric cancer patients had available follow-up data on outcome. The overall survival time was calculated from the date of registration at M.D. Anderson to the date of last contact or death. Patients who were still alive at the last contact were considered as a censored event in the analysis. The age at diagnosis, sex, and type of treatments (i.e., surgery and chemotherapy) were used as covariates in the analysis. The age at diagnosis was categorized into two groups according to the mean age (≤ 57 and >57 years).

### Statistical Analysis

Two-sided chi-square and *t *tests were performed to determine any statistically significant differences in the distributions of categorical variables (e.g., the *TGFB1 *and *VEGF *alleles and genotypes) by demographic variables and clinical features and in the means of continuous variables (e.g., age and survival time), respectively. The distributions of the genotypes were tested for deviation from Hardy-Weinberg equilibrium (HWE), and the haplotypes for the variants of the same gene were reconstructed according to the PHASE program [9], by which each individual's probability of having a particular haplotype pair was estimated, and the haplotype pair with the highest estimated probability was assigned to the individual. Pearson's chi-square or global test was used to test for the survival differences among patients by all haplotypes. Overall survivals among the three genotype groups of each SNP were analyzed using the Kaplan-Meier method, and the log-rank test was used to test for the equality of the survival distributions stratified by genotypes. We used univariate and multivariate Cox proportional hazards models to estimate the effect of each genotype on survival in the presence of other covariates. Both age at diagnosis and the time interval between registration and diagnosis date (pathologic confirmation of disease) were treated as numeric covariates in the Cox model. To confirm the assumption of proportional hazards in a Cox regression model, we added a time-dependent variable to the model, and the assumption was confirmed. Hazard ratios (HRs) and their corresponding 95% confidence intervals (CIs) were calculated with adjustment for other covariates in the same model. The joint effects of the *TGFB1 *and *VEGF *SNPs and their interactions with smoking and drinking on gastric cancer risk were also evaluated. All statistical tests were 2-sided, with a *P *value of 0.05 considered significant and all were calculated using SAS software (version 9.1; SAS Institute, Cary, NC).

## Results

### Characteristics of the study population

Clinical and pathological characteristics of the 167 patients enrolled in this study are shown in Table 1. There were 114 males (68.3%) and 53 females (31.7%), whose ages ranged from 32 to 89 years. Using the Cox regression analysis of the relationship between overall survival and clinicopathologic characteristics, we found that neither age, gender, ethnicity, smoking, nor alcohol status were statistically associated with overall survival (*P *= 0.339, 0.988, 0.297, 0.475, 0.809, respectively).

**Table 1:** Characteristics of the study population of patients with gastric cancer

**Characteristics**	**No. of Patients**	**No. of Deaths**	**MST (months)**	P*
**Total subjects** **Age (mean)**	167	60		0.339
≤57 years	68	27	21.2	
>57 years	99	33	31.0	
**Gender**				0.988
Male	114	41	23.3	
Female	53	19	28.9	
**Ethnicity**				0.297
White	117	45	28.8	
Non-White^†^	50	15	19.1	
**Smoke**				0.475
Never	34	14	20.6	
Ever	133	46	30.1	
**Alcohol**				0.809
Never	62	23	23.2	
Ever	105	37	29.3	
**Location**				0.069
Stomach	118	36	24.3	
Esophagus	25	13	27.2	
GEJ	24	11	16.6	
**Histology**				0.356
Intestinal	118	45	28.1	
Signet ring	49	15	24.6	
**Differentiation**				0.694
Poor	96	37	21.8	
Moderate-poor	28	10	29.8	
Moderate-Well	42	13	22.6	
**Clinical Stage**				**< 0.001**
I + II	65	9	30.4	
III + IV	101	51	22.7	
**Metastasis**				**< 0.001**
yes	90	49	21.2	
no	77	11	34.2	
**Chemotherapy**				**< 0.001**
yes	121	54	26.3	
no	46	6	10.4	
**Surgery**				**< 0.001**
yes	63	11	39.2	
no	104	49	18.4	

Abbreviations: MST, median survival time; GEJ, gastroesophageal junction.

* Chi-square test.

^†^Included 13 Asians, 16 blacks, 19 Hispanics, and 2 Native Indians.

The tumors of 118 (70.7%) the patients were located at the stomach and those of 49 (29.3%) patients were located at the gastroesophageal junction (GEJ). Regardless of tumor location, all the patients had adenocarcinoma. Of these, 118 (70.7%) patients were intestinal and 49 (29.3%) signet ring. We grouped the types of differentiation into the following three categories: poor, moderate-poor and moderate-well, and the number and percentage of these three groups were 96 (57.8%), 28 (16.9%) and 42 (25.3%), respectively. In all patients, clinico-pathological characteristics including tumor location, histology and differentiation status were not significantly associated with overall survival in the univariate analysis (*P *= 0.069, 0.356, and 0.694, respectively). Clinical tumor stages according to the International Union Against Cancer (UICC) criteria were as follows: 65 (38.9%) had stage I+II and 101 (61.1) had stage III +IV (Table 1).

Among the 167 patients, 121 (72.4%) received chemotherapy, and 63 (37.7%) received surgery; at the end of the follow-up period, 60 (35.9%) patients had died. The mean follow-up time was 18.0 ± 13.3 months for the patients who were still alive, and the mean survival time for all patients was 29.4 months. Advanced stage, metastasis, chemotherapy and surgery were all associated with overall survival (*P *< 0.001 for all) (Table 1). For example, the mean survival time was 34.2 months for patients without metastasis and 21.2 months for those with metastasis. Those who received chemotherapy and surgery had a longer mean survival time than those who did not (26.3 months versus 10.4 months for chemotherapy and 39.2 months versus 18.4 months for surgery).

### HWE, linkage disequilibrium and haplotypes TGFB1 and VEGF

For *TGFB1*, one of the three SNPs (rs1800469C>T, rs1800470T>C and rs1800471G>C) was not in HWE (*P *< 0.05 for rs1800469C>T), suggesting a possible selection bias, but none of the *VEGF *SNPs (rs833061T>C, rs2010963G>C and rs3025039C>T) departed from HWE (*P *> 0.05 for all). None of the pairs of *TGFB1 *or *VEGF *SNPs were in high linkage disequilibrium (i.e., r^2 ^between 0.039 and 0.541, all <0.08). Only four *TGFB1 *haplotypes and five *VEGF *haplotypes had an allele frequency of >0.05 (C-T-G, 0.570; C-C-G, 0.190; T-C-G, 0.167 and C-C-C, 0.063 for *TGFB1 *and C-G-C, 0.344; T-C-C, 0.287; T-G-C, 0.192; C-G-T, 0.072 and T-C-T, 0.051 for *VEGF*). Because of the small sample size, we did not calculate the diplotypes.

### TGFB1 and VEGF genotype distributions and overall survival

When all gastric cancer patients were analyzed for overall survival, no significant difference was found in the distributions of mean survival time by genotypes for any of the polymorphisms studied. Because there were few participants in the minor homozygous variant groups, we combined the heterozygous and minor variant homozygous genotypes together for additional analysis, assuming a dominant genetic model, but there was still no association between detected polymorphisms and overall survival (see Additional file 1). Furthermore, when the gastric cancer patients were stratified by age, sex, ethnicity, and metastatic status, no difference in the distribution according to mean survival time by the six SNPs was found among the subgroups (see Additional file 1).

### TGFB1 and VEGF genotype distributions and 1-and 2-year survivals

**Figure 1:**
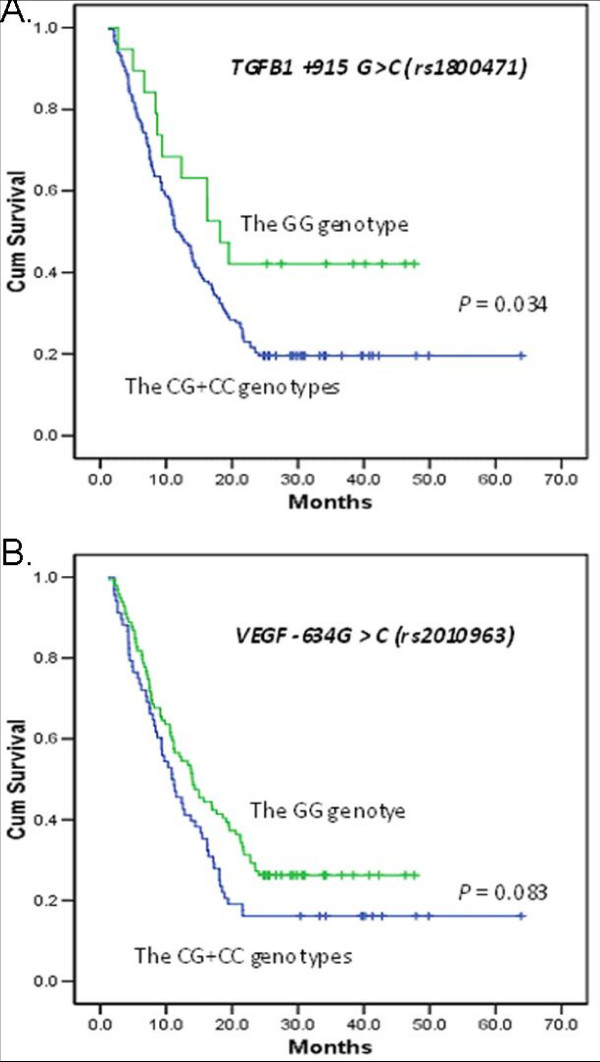
**Cumulative survival functions of the genotypes *TGFB1 *+915 G>C (rs1800471) and *VEGF *-634G>C (rs2010963)**.

Because the prognosis is generally poor in advanced cases of gastric cancer, median survival rarely approaches 1 or 2 years [2]. In the present study, most of the cases were stage IV (101/167) with a median survival time of only 16.2 months (95% CI, 12.8–24.9). Therefore, we also calculated the 1-year and 2-year survival rates for patients with different genotypes (see Additional file 2). The overall 1-year and 2-year survivals for all patients were 51.5% and 22.1%, respectively. Although there were no significant differences in the survival rates between most genotypes, patients with *TGFB1 +*915CG/CC genotypes had better 1-year and 2-year survival than those with the GG genotype (adjusted HR, 2.13; 95% CI, 0.76–6.01; *P *= 0.122 and adjusted HR, 3.06; 95% CI, 1.09–8.62; *P *= 0.034, respectively) (Figure 1). Furthermore, patients heterozygous for *VEGF *-634CG also had a better 1-year survival rate (adjusted HR, 2.08; 95% CI, 1.03–4.22; *P *= 0.042) than those with the *VEGF *-634 GG genotype.

Further analyses combining the alleles, genotypes, and haplotypes of the same gene did not substantiate the findings from the single locus analysis (data not shown), partly because of the low LD among the SNPs and the small study size that did not allow for further stratification analysis.

## Discussion

The etiology of gastric cancer is multifactorial, multigenetic and multistage [24,25]. It is known that during carcinogenesis, TGF-β can switch from a tumor suppressor to a tumor enhancer in the later stages of cancer [26]. With dual role in cancer development, there is great interest in analyzing the role of genetic variation in *TGFB1 *in cancer progression and patient survival. For example, the *TGFB1 *-509C>T and rs1982073 (or rs1800470) polymorphisms have been shown to be associated with breast cancer survival in a Chinese population [27-30] and chemoradiotherapy response in 175 Finnish patients with head and neck squamous cancer[31], respectively. However, neither *TGFB1 *+869T>C nor +915G>C polymorphisms showed any association with tumor relapse and progression in bladder tumors without muscular invasive in a Spanish population [32]. While a Korean study showed that the variant T genotypes of the *TGFB1 *-509C>T SNP were associated with a reduced risk of lung cancer [33], a Chinese study of 414 patients and 414 controls [34] reported that the genotypes were not associated with an overall risk of developing gastric cancer but with a decreased risk of risk of stage I or II gastric cancer. However, no survival analyses were presented in these studies.

As noted, we did not find any statistical evidence to support a significant association between *TGFB1*polymorphisms and overall survival in gastric cancer. However, the significant association between *TGFB1+*915 CG/CC genotypes and 2-year survival for all gastric cancer patients suggests that this *TGFB1*variant may have attenuated the role of TGF-β1 as a tumor suppressor in the earlier stage of tumor progression. It is also known that TGF-β1 can switch from a tumor suppressor to a tumor enhancer in the late stage of cancer [26]. Once the tumors had grown bigger and become metastatic, the resultant increase in somatic mutations or gains in the copies of oncogenes may have outweighed the role of the suppressor variants in the late stages of the tumor, leading to no difference in overall survival of the patients with different genotypes of the *TGFB1+*915 G>C SNP. However, this speculation needs to be validated in more rigorously designed studies with a much larger sample size and more information on the mutation spectrum in the tumors.

VEGF, as a key mediator of angiogenesis, also plays an important role in the development of cancers. *VEGF *polymorphisms have also been shown to be associated with survival in both gastric cancer and colorectal cancer [35,36]. However, the results from published studies remain inconsistent rather than conclusive. In a Greek gastric cancer study of five *VEGF *SNPs (-2578C>A, -1154G>A, -634G>C, -460T>C, and +936C>T) in 312 patients [36], *VEGF *-2578 AA, -634 CC and +936TT genotypes were associated with a significantly lower HR (better survival) for 6-year survival of colorectal cancers. Interestingly, another early Greek study of 100 gastric cancer patients suggested that only the *VEGF *-634CC/CG genotypes were associated with a decreased (poorer survival) 10-year survival, compared with the GG genotype [35]. Our data on 167 gastric cancer patients indicated that *VEGF *-634CC/CG carriers indeed had a poor 1-year survival than those with the *VEGF *-634 GG genotype. Amano et al. [37] also reported that no significant association was observed between the frequencies of the *VEGF *-460T>C, +405G>C, and 936C>T genotypes and 3-year disease-free survival of endometrial carcinoma patients in a Japanese study of 105 endometrial carcinoma patients. Because all these studies, including ours, have been relatively small, there was limited ability to perform the more powerful haplotype-based analysis that the analysis of a single allele or locus effect [34].

This is the first report, to our knowledge, involving *TGFB1 *and *VEGF *polymorphisms and survival in gastric cancer patients mainly consisting of a Caucasian population; however, there were some limitations to the present study. Although we tried to collect recurrence data on these patients, we could not investigate this end-point due to the lack of a pre-defined follow-up plan. A second limitation was the fact that we only included three common *TGFB1 *SNPs and three *VEGF *SNPs. It is possible that some other important SNPs were missed or that the observed associations may be due to other polymorphisms in LD with the SNPs we studied. Also, no data on serum/plasma protein levels were available for the genotype-phenotype correlation analysis, because only DNA samples were available from these patients. There are other genes in addition to *TGFB1 *and *VEGF *that also play a role in cell growth and angiogenesis, representing a complex interplay of many activating and inhibitory factors [38]. Furthermore, *Helicobacter pylori *infection, the presence or absence of which was not reported in the present study, is considered to be the cause of a progressive accumulation of genotypic changes in gastric cancer, which may lead to sporadic gastric cancer carcinogenesis [39]. Finally, the study size was too small to have a sufficient power to detect small HRs. For example, our post-power calculation suggested that the sample size for an equal number (n = 55) of subjects in each genotype of each SNP, the power to detect an HR of 2 was <0.4, but >0.8 for a HR of 3.4 for a follow-up time of 5 years. Therefore, only the finding of HRs for 2-year survival of *TGFB1 *+915G>C would have a sufficient power, suggesting a much larger study would be needed to effectively test our hypothesis for effects of the overall survival.

## Conclusion

In summary, we found that some polymorphisms *TGFB1 *and *VEGF *may be associated with 1- or 2-year survival rates of gastric cancer patients. However, the present study is small, and various genetic and epigenetic events may also have led to an association between *TGFB1 *and *VEGF *polymorphisms and gastric cancer prognosis and survival. Therefore, larger and better-designed studies are required to overcome the limitations in the present study (particularly the information about *Helicobacter pylori *infection) and further confirm our observations.

## Abbreviations

*TGF-β1*: Transforming growth factor beta 1; *VEGF*: Vascular endothelial growth factor; LD: linkage disequilibrium; PCR-RFLP: polymerase chain reaction-restriction fragment length polymorphism; OR: odds ratio; HR: hazard ratio; CI: confidence interval; SNP: single nucleotide polymorphism.

## Competing interests

The authors declare that they have no competing interests.

## Authors' contributions

XG performed the laboratory work, acquisition of data, and drafted the manuscript. HZ performed statistical analysis and read the manuscript. JN assisted in performing laboratory work, statistical analysis and proofreading of the manuscript. DT and JAA performed the patient and pathological evaluation and read the manuscript. QW conceived and coordinated the study, checked statistical results, read and edited the manuscript. All the authors read and approved the final manuscript.

## Supplementary Material

Additional file 1 **TGFB1 and VEGF genotype distributions and overall survival**. The data provided represent the statistical analysis of TGFB1 and VEGF genotype distributions and overall survival. Click here for file

Additional file 2 **TGFB1 and VEGF genotype distributions and 1-and 2-year survivals**. The data provided represent the statistical analysis of TGFB1 and VEGF genotype distributions and 1-and 2-year survivals. Click here for file
