# Supplementary material for: Polymorphisms of TGFB1 and VEGF genes and survival of patients with gastric cancer
Source: J Exp Clin Cancer Res. 2009 Jun 30;28(1):94. doi: 10.1186/1756-9966-28-94 (PMC2717936; doi:10.1186/1756-9966-28-94)
Supplement: Additional file 1 — TGFB1 and VEGF genotype distributions and overall survival. The data provided represent the statistical analysis of TGFB1 and VEGF genotype distributions and overall survival. [file 1756-9966-28-94-S1.doc]

| **Additional File 1. Association between *TGFB1* and *VEGF* polymorphisms and overall survival in patients with gastric cancer** | | | | | |
| --- | --- | --- | --- | --- | --- |
| **Genotypes** | **No. Of Patients** | **No. of Deaths** | **MST**  **(months)*** | **Hazard Ratio (95% CI)** | ***P*** ^†^ |
| ***TGFB1 -509 C >T (rs1800469)*** | | |  |  |  |
| CC | 121 | 49 | 27.9±1.9 | 1.00 |  |
| CT | 33 | 7 | 27.7±2.5 | 0.47 (0.21-1.06) | 0.068 |
| TT | 13 | 4 | 14.7±1.7 | 0.82 (0.29-2.30) | 0.706 |
| CT+TT | 46 | 11 | 26.1±2.2 | 0.55 (0.28-1.09) | 0.089 |
| ***TGFB1 +869 T >C (rs1800470)*** | | |  |  |  |
| TT | 57 | 21 | 20.2±1.6 | 1.00 |  |
| CT | 80 | 28 | 30.0±2.4 | 0.75 (0.42-1.35) | 0.342 |
| CC | 30 | 11 | 19.0±1.5 | 0.79 (0.38-1.68) | 0.542 |
| CT+CC | 110 | 39 | 29.0±1.8 | 1.07 (0.55-2.09) | 0.839 |
| ***TGFB1 +915 G >C (rs1800471)*** | | |  |  |  |
| GG | 148 | 52 | 23.4±1.2 | 1.00 |  |
| CG | 17 | 7 | 32.9±4.6 | 0.77 (0.35-1.73) | 0.532 |
| CC | 2 | 1 | 9.4±0.0 | 3.10 (0.41-23.6) | 0.274 |
| CG+CC | 19 | 8 | 31.6±4.5 | 0.86 (0.40-1.83) | 0.685 |
| ***VEGF -1498T > C (rs833061)*** | | |  |  |  |
| TT | 50 | 15 | 19.6±1.3 | 1.00 |  |
| CT | 80 | 30 | 29.2±2.4 | 1.14 (0.60-2.14) | 0.697 |
| CC | 37 | 15 | 20.4±2.4 | 1.79 (0.85-3.76) | 0.123 |
| CT+CC | 117 | 45 | 27.6±2.0 | 1.29 (0.71-2.35) | 0.412 |
| ***VEGF -634G > C (rs2010963)*** | | |  |  |  |
| GG | 68 | 26 | 22.3±1.8 | 1.00 |  |
| CG | 70 | 22 | 31.9±2.5 | 0.69 (0.39-1.22) | 0.204 |
| CC | 29 | 2 | 19.9±2.3 | 1.11 (0.55-2.23) | 0.765 |
| CG+CC | 99 | 24 | 30.3±2.1 | 0.79 (0.47-1.34) | 0.384 |
| ***VEGF +936C > T (rs3025039)*** | | |  |  |  |
| CC | 124 | 44 | 29.0±1.9 | 1.00 |  |
| CT | 40 | 14 | 17.8±1.3 | 0.98 (0.53-1.80) | 0.937 |
| TT | 3 | 2 | 10.4±2.2 | 3.12 (0.72-13.7) | 0.130 |
| CT+TT | 43 | 16 | 17.5±1.2 | 1.07 (0.59-1.91) | 0.831 |
| Abbreviation: MST, mean survival time.  ^*^The MST was underestimated because the largest observation was censored and the estimation was restricted to the largest even time.  ^†^ P values were calculated using the log-rank test and adjusted for age, gender, smoking status and alcohol status. | | | | | |
