# Supplementary material for: Polymorphisms of TGFB1 and VEGF genes and survival of patients with gastric cancer
Source: J Exp Clin Cancer Res. 2009 Jun 30;28(1):94. doi: 10.1186/1756-9966-28-94 (PMC2717936; doi:10.1186/1756-9966-28-94)
Supplement: Additional file 2 — TGFB1 and VEGF genotype distributions and 1-and 2-year survivals. The data provided represent the statistical analysis of TGFB1 and VEGF genotype distributions and 1-and 2-year survivals. [file 1756-9966-28-94-S2.doc]

| **Table 3. Association between *TGFB1* and *VEGF* variants and 1- and 2- year survival rates in patients with gastric cancer** | | | | | | | | | | | | |
| --- | --- | --- | --- | --- | --- | --- | --- | --- | --- | --- | --- | --- |
| Variables | 1- year survival rate | | | | HR (95% CI)* | *P*^†^ | 2- year survival rate | | | | HR (95% CI)* | *P*^†^ |
|  | >1 year | | ≤1 year | |  |  | >2 years | | ≤2 years | |  |  |
|  | n | % | n | % |  |  | n | % | n | % |  |  |
| ***TGFB1 -509 C >T (rs1800469)*** | | | |  |  |  |  |  |  |  |  |  |
| CC | 64 | 74.4 | 57 | 70.4 | 1.00 |  | 29 | 78.4 | 92 | 70.8 | 1.00 |  |
| CT | 16 | 18.6 | 17 | 21.0 | 0.92 (0.42-2.03) | 0.837 | 7 | 18.9 | 26 | 20.0 | 0.93 (0.35-2.45) | 0.878 |
| TT | 6 | 7.0 | 7 | 8.6 | 0.83 (0.26-2.66) | 0.749 | 1 | 2.7 | 12 | 9.2 | 0.28 (0.03-2.29) | 0.295 |
| CT/TT | 22 | 25.6 | 24 | 29.6 | 0.89 (0.45-1.79) | 0.750 | 8 | 21.6 | 38 | 29.2 | 0.72 (0.29-1.76) | 0.465 |
| ***TGFB1 +869 T >C (rs1800470)*** | | | |  |  |  |  |  |  |  |  |  |
| TT | 25 | 29.1 | 32 | 39.5 | 1.00 |  | 11 | 29.7 | 46 | 35.4 | 1.00 |  |
| CT | 45 | 52.3 | 35 | 43.2 | 1.75 (0.85-3.62) | 0.129 | 21 | 56.8 | 59 | 45.4 | 1.69 (0.70-4.12) | 0.244 |
| CC | 16 | 18.6 | 14 | 17.3 | 1.53 (0.62-3.80) | 0.365 | 5 | 13.5 | 25 | 19.2 | 0.84 (0.25-2.79) | 0.775 |
| CT/CC | 25 | 29.1 | 49 | 60.5 | 1.68 (0.86-3.31) | 0.320 | 26 | 70.3 | 84 | 64.6 | 1.40 (0.60-3.23) | 0.434 |
| ***TGFB1 +915 G >C (rs1800471)*** | | | |  |  |  |  |  |  |  |  |  |
| GG | 73 | 84.9 | 75 | 92.6 | 1.00 |  | 29 | 78.4 | 119 | 91.6 | 1.00 |  |
| CG | 13 | 15.1 | 4 | 4.9 | 3.32 (1.01-10.8) | **0.047** | 8 | 21.6 | 9 | 6.9 | 3.91 (1.33-11.5) | **0.013** |
| CC | 0 | 0 | 2 | 2.5 | -- | -- | 0 | 0 | 2 | 1.5 | -- | -- |
| CG/CC | 13 | 15.1 | 6 | 7.4 | 2.13 (0.76-6.01) | 0.152 | 8 | 21.6 | 11 | 8.4 | 3.06 (1.09-8.62) | **0.034** |
| ***VEGF -1498T > C (rs833061)*** | | | |  |  |  |  |  |  |  |  |  |
| TT | 27 | 31.4 | 23 | 28.4 | 1.00 |  | 13 | 35.1 | 37 | 28.5 | 1.00 |  |
| CT | 44 | 51.2 | 36 | 44.4 | 1.06 (0.51-2.21) | 0.877 | 19 | 51.4 | 61 | 46.9 | 0.99 (0.42-2.30) | 0.976 |
| CC | 15 | 17.4 | 22 | 27.2 | 0.49 (0.20-1.21) | 0.122 | 5 | 13.5 | 32 | 24.6 | 0.41 (0.13-1.31) | 0.131 |
| CT/CC | 59 | 68.6 | 58 | 71.6 | 0.84 (0.42-1.68) | 0.625 | 24 | 64.9 | 93 | 71.5 | 0.77 (0.35-1.72) | 0.523 |
| ***VEGF -634G > C (rs2010963)*** | | | |  |  |  |  |  |  |  |  |  |
| GG | 31 | 36.1 | 37 | 45.7 | 1.00 |  | 11 | 29.7 | 57 | 43.9 | 1.00 |  |
| CG | 42 | 48.8 | 28 | 34.6 | 2.08 (1.03-4.22) | **0.042** | 18 | 48.7 | 52 | 40.0 | 2.01 (0.84-4.81) | 0.117 |
| CC | 13 | 15.1 | 16 | 19.7 | 1.06 (0.43-2.62) | 0.899 | 8 | 21.6 | 21 | 16.1 | 2.18 (0.74-6.42) | 0.158 |
| CG/CC | 55 | 63.9 | 44 | 54.3 | 1.70 (0.89-3.25) | 0.108 | 26 | 70.3 | 73 | 56.1 | 2.06 (0.91-4.66) | 0.083 |
| ***VEGF +936C > T (rs3025039)*** | | | |  |  |  |  |  |  |  |  |  |
| CC | 60 | 69.8 | 64 | 79.0 | 1.00 |  | 29 | 78.4 | 95 | 73.1 | 1.00 |  |
| CT | 24 | 27.9 | 16 | 19.8 | 1.56 (0.74-3.27) | 0.243 | 8 | 21.6 | 32 | 24.6 | 0.79 (0.32-1.96) | 0.615 |
| TT | 2 | 2.4 | 1 | 1.2 | 1.84 (0.16-21.3) | 0.627 | 0 | 0 | 3 | 2.3 | -- | 0.981 |
| CT/TT | 26 | 30.1 | 17 | 21 | 1.57 (0.76-3.25) | 0.221 | 8 | 21.6 | 35 | 26.9 | 0.73 (0.30-1.79) | 0.487 |
| * Hazard ratio was adjusted for age, gender, smoking status and alcohol use. ^†^Two-sided chi-square test for difference between frequency distribution | | | | | | | | | | | | |
